# Characterization of antibody response to SARS-CoV-2 Orf8 from three waves of COVID-19 outbreak in Thailand

**DOI:** 10.1371/journal.pone.0297272

**Published:** 2024-05-20

**Authors:** Jeeraphong Thanongsaksrikul, Paskorn Sritipsukho, Potjanee Srimanote, Onruedee Khantisitthiporn, Wipawadee Sianglum, Uayporn Pinitchai, Yong Poovorawan

**Affiliations:** 1 Graduate Program in Biomedical Sciences, Faculty of Allied Health Sciences, Thammasat University, Pathumthani, Thailand; 2 Thammasat University Research Unit in Molecular Pathogenesis and Immunology of Infectious Diseases, Thammasat University, Pathumthani, Thailand; 3 Healthcare Service Center, Faculty of Allied Health Sciences, Thammasat University, Pathumthani, Thailand; 4 Center of Excellence in Applied Epidemiology, Thammasat University, Pathumthani, Thailand; 5 Department of Pediatrics, Faculty of Medicine, Thammasat University, Pathumthani, Thailand; 6 Department of Medical Technology, Faculty of Allied Health Sciences, Thammasat University, Pathumthani, Thailand; 7 Division of Biological Science, Faculty of Science, Prince of Songkla University, Songkhla, Thailand; 8 Thammasat University Hospital, Thammasat University, Pathumthani, Thailand; 9 Center of Excellence in Clinical Virology, Department of Pediatrics, Faculty of Medicine, Chulalongkorn University, Bangkok, Thailand; Nuclear Science and Technology Research Institute, ISLAMIC REPUBLIC OF IRAN

## Abstract

A dynamic of virus adaptation and a mass vaccination campaign could significantly reduce the severity of clinical manifestations of COVID-19 and transmission. Hence, COVID-19 may become an endemic disease globally. Moreover, mass infection as the COVID-19 pandemic progressed affected the serology of the patients as a result of virus mutation and vaccination. Therefore, a need exists to acquire accurate serological testing to monitor the emergence of new outbreaks of COVID-19 to promptly prevent and control the disease spreading. In this study, the anti-Orf8 antibodies among samples collected in Thailand’s first, fourth, and fifth waves of COVID-19 outbreaks compared with pre-epidemic sera were determined by indirect ELISA. The diagnostic sensitivity and specificity of the anti-Orf8 IgG ELISA for COVID-19 samples from the first, fourth, and fifth waves of outbreaks was found to be 100% compared with pre-epidemic sera. However, the diagnostic sensitivity and specificity of the anti-Orf8 IgG ELISA for a larger number of patient samples and controls from the fifth wave of outbreaks which were collected on day 7 and 14 after an RT-PCR positive result were 58.79 and 58.44% and 89.19 and 58.44%, respectively. Our data indicated that some of the controls might have antibodies from natural past infections. Our study highlighted the potential utility of anti-Orf8 IgG antibody testing for seroprevalence surveys but still warrants further investigations.

## Introduction

In 2019, severe acute respiratory syndrome coronavirus 2 (SARS-CoV-2) was discovered as a novel human coronavirus causing severe respiratory syndrome, namely, coronavirus disease 2019 (COVID-19) [1]. The first case of laboratory-confirmed COVID-19 outside China was reported from Thailand on 13 January 2020, namely, an individual aged 61 years traveling from Wuhan by direct flight to Thailand [2]. Later on, an additional 14 imported cases from China were detected [3]. The first Thai national case was reported on 31 January 2020, namely, a taxi driver who contracted the disease from tourist passengers. From February to March, new COVID-19 cases continued to increase ranging from one to 11 cases daily [2]. Local transmission of COVID-19 was limited and most new cases were primarily travel-related and confined to Bangkok [2]. Until 15 March, the new cases sharply increased to over 100 cases daily [2]. By the end of March, 60 of 77 provinces had reported cases and the epidemic of COVID-19 was confirmed in Thailand [2, 3]. From 2020 to 2022, Thailand experienced five waves of COVID-19 outbreaks as described by Puenpa J. et al. 2022 [4]. The first outbreak of COVID-19 in Thailand was considered to be clusters of people attending boxing events and entertainment venues in Bangkok from March to May 2020. As episodes of the epidemic continued, the second to fifth waves were reported from December 2020 to February 2021, April to June 2021, July to December 2021, and January to March 2022, respectively [4]. Each wave of COVID-19 outbreaks in Thailand was predominated by different epidemic clades and strains of SARS-CoV-2 [4]. Clade S (lineages A and B.1) was predominant in COVID-19 cases of Thailand’s first wave. However, most COVID-19 cases in the second to fifth waves were caused by clade GH B.1.36.16, clade GRY B.1.1.7 (alpha variant), clade GK B.1.617.2 (delta variant) and clade GRA B.1.1.529 (omicron variant), respectively. The rapid emergence of new SARS-CoV-2 variants is mainly caused by mutations in the virus genome facilitated by RNA-dependent RNA polymerase lacking proof-reading activity [5]. The mutations are attributed to virus adaptations such as enhanced transmissibility, altered virulence, and immune evasion [5]. Moreover, the virus mutations could compromise vaccine and antiviral drug effectiveness as well as the accuracy of diagnosis.

A dynamic of virus adaptation and a mass vaccination campaign could significantly reduce the severity of clinical manifestations of COVID-19 and transmission. Mass infection from epidemic episodes affected by multiple SARS-CoV-2 variants could allow the natural selection of evolutionarily adapted viruses that have the fitness to gain higher transmissibility but are compromised in their pathogenic ability to become predominant epidemic strain [6]. It has been shown that the omicron-dominant epidemic waves have a higher transmissibility rate and less clinical severity than those of the delta-dominant waves [7]. Hence, COVID-19 seems to have become an endemic disease globally. In this situation, the need exists to acquire a diagnostic tool to monitor the emergence of new outbreaks of COVID-19 and to promptly prevent and control the disease from spreading. Serological testing could be used as a complementary test of PCR-based methods for COVID-19 diagnosis. It offers advantages over PCR after two weeks of disease onset at which the efficiency of the latter test declines [8]. Moreover, it constitutes a valuable tool to evaluate immune responses to natural infections and vaccines. Current serological testing for COVID-19 is aimed at measuring antibodies, either IgM or IgG, specific to viral proteins including spike (S) and nucleocapsid (N) [9]. Even though the detection of anti-S and anti-N antibodies has shown high diagnostic accuracy for COVID-19, it could not distinguish between antibodies generated in response to vaccine and infection. Natural infection stimulates the humoral immune response to structural, nonstructural, and accessory SARS-CoV-2 proteins [10]. The vaccination with current COVID-19 vaccines mostly targets the production of neutralizing anti-S antibodies except for killed/attenuated vaccines which stimulate the production of antibodies to multiple proteins particularly structural proteins including S, M, E, and N [11]. Therefore, alternative viral antigens should be explored to develop serological testing of COVID-19 capable of detecting antibodies exclusively generated in response to infection. Therefore, SARS-CoV-2 accessory and nonstructural proteins should be the best candidates because they could not be found in the current COVID-19 vaccine component and are produced only in the infected cells during viral replication [12].

Orf8 is one of the accessory proteins of SARS-CoV-2 [13]. It has been reported that the amino acid sequence of Orf8 is conserved in SARS-CoV-2 and has low homology to SARS-CoV-1 and common human coronaviruses [13]. Orf8 could be secreted from transfected cells [14] and is found in the serum of patients with COVID-19 [15]. Moreover, Orf8 is an immunodominant antigen and an anti-Orf8 antibody has been reported to be an accurate serological marker of SARS-CoV-2 [16, 17]. However, the *Orf8* coding sequence possesses a hypervariable genomic region that undergoes rapid nucleotide substitutions and deletions during epidemics and is affected by different variants of SARS-CoV-2 [18]. This issue might lead to reduced diagnostic accuracy in detecting anti-Orf8 antibodies. Nevertheless, no reports exist concerning this matter. Therefore, in this study, antibody response to SARS-CoV-2 Orf8 was characterized and compared among COVID-19 sera collected from three waves of Thailand’s outbreak. The gained information will elucidate the utility of anti-Orf8 antibody detection as serological testing of COVID-19 in the post-pandemic and post-vaccination era. The details of the methods and results are mentioned herein.

## Materials and methods

### Samples

#### Pre-epidemic samples

As COVID-19-free samples, a total of 21 serum samples were obtained from healthy blood donors at Songklanagarind Hospital, Faculty of Medicine, Prince of Songkla University, Thailand, in January 2020. The samples were screened for infectious markers including HIV-1, HCV, and HBV by nucleic acid testing. The samples were stored at -40°C until use. All samples were determined for neutralizing antibodies specific to the S protein of SARS-CoV-2 using a surrogate virus neutralization assay (GenScript cPass™ SARS-CoV-2 Neutralization Antibody Detection Kit).

#### Samples collected from the first and fourth waves of COVID-19 outbreaks in Thailand

The samples of the first wave of COVID-19 outbreaks (March to May 2020) included a total of 60 convalescence plasma samples collected from 60 individuals who had recovered from COVID-19 and donated their plasma to the Thai Red Cross Society. From the fourth wave of COVID-19 outbreaks, a total of 62 serum samples were collected from 38 patients with COVID-19 who had been hospitalized at Thammasat University Hospital in Thailand from July to December 2021. COVID-19 was diagnosed using real-time reverse-transcription polymerase chain reaction (real-time RT-PCR).

#### Samples collected from the fifth wave of COVID-19 outbreak in Thailand

The samples of the fifth wave of COVID-19 outbreaks were collected at Thammasat University Hospital in Thailand from January to June 2022. A total of 327 serum samples were collected from 216 patients receiving a diagnosis of COVID-19 using real-time RT-PCR (case group). In total, 105 sera samples were collected once from individual patients seven days after PCR tested positive (visit 1). One hundred eleven patients were collected twice for serum at 7 (visit 1) and 14 days after PCR tested positive (visit 2). A total of 669 serum samples were collected once from individual patients hospitalized at the Thammasat University Hospital and clinically diagnosed by their doctors as having a disease other than COVID-19 as well as testing negative for SARS-CoV-2 using real-time RT-PCR (control group).

All samples of the three waves of outbreaks were collected at least 7 to 14 days after the disease onset and stored at -40°C until use. Antibodies specific to N and S proteins of SARS-CoV-2 (anti-N and anti-S IgG) of all samples were verified using ELISA.

### Clinical data

Patient information including vaccination history and clinical severity of COVID-19 was collected retrospectively from hospital electronic records. The clinical severity of COVID-19 among patients was graded according to CDC guidelines [19].

### Development of ELISA to detect IgG antibodies to Orf8 of SARS-CoV-2 and detection of anti-Orf8 IgG in samples collected from the outbreaks in Thailand

Conditions of indirect ELISA to detect anti-Orf8 IgG antibodies were optimized using known COVID-19 positive samples, and pre-epidemic serum samples as negative controls were applied in the subsequent experiments. To immobilize the antigen, a mixture of 12.5 ng of Orf8 recombinant protein (Thermo Fisher Scientific, Cat. No. RP-87666) in 100 μl carbonate buffer pH. 9.6 was added to each well of the ELISA microplate and incubated at 37°C for 16 to 18 hours. The wells were washed with phosphate-buffered saline containing 0.05% Tween-20 (PBS-T). The empty surface areas were blocked with BlockPRO™ protein-free blocking buffer (Visual Protein, Taiwan). Serum samples were 1:10,000 diluted in a blocking buffer. The diluted samples were incubated with the immobilized Orf8 antigen at 37°C for 1 hour. The wells were washed and incubated with mouse anti-human IgG antibody followed by goat anti-mouse immunoglobulin antibody conjugated with horseradish peroxidase (HRP). Then 3,3’,5,5’-Tetramethylbenzidine (TMB) chromogenic substrate was added to the wells and incubated at 37°C for 15 minutes. The enzymatic reaction was terminated by adding 0.1 M sulfuric acid stop solution to the wells. The generated color signals were measured by spectrophotometer (Biotek) using optical density at 450 nm (OD_450nm_) subtracted by OD_570nm_ (wavelength correction). The pre-epidemic sample that gave the highest OD value was selected to use as a calibrator of the subsequent ELISA testing. Then ratios between each sample’s OD values to the calibrator’s OD value (S/C) were calculated [20].

### Statistical analysis

The ELISA OD values of all samples were compared with those of the calibrator and expressed as a ratio of sample to calibrator (S/C) signal. The optimal S/C cut-off value for anti-Orf8 IgG ELISA was determined employing receiver operating characteristic (ROC) curve analysis and used to diagnose the samples. The diagnostic accuracy of the anti-Orf8 IgG ELISA was determined by the 2 × 2 table method using the samples from the fifth wave of COVID-19 outbreaks. Agreement between real-time RT-PCR and anti-Orf8 IgG ELISA was analyzed by weighted Kappa using inter-rater agreement statistics. Means and standard deviations (s.d.) of S/C values of the independent groups were calculated. The comparisons between S/C values of COVID-19 convalescent sera and pre-epidemic sera or control group were analyzed using the t-test. The S/C values among three independent groups of COVID-19 convalescent sera were compared using one-way analysis of variance (one-way ANOVA) with Tukey-Kramer pair-wise comparison as necessary. The comparison of anti-Orf8 IgG levels of paired samples was analyzed by paired sample t-test. A *p*-value less than 0.05 is considered a statistically significant difference. MedCalc^®^ Statistical Software, Version 22.014 was used for all analyses (MedCalc Software Ltd, Ostend, Belgium).

## Results

### Sensitivity and specificity of the ELISA SARS-CoV-2 Orf8 assay

**Fig 1:**
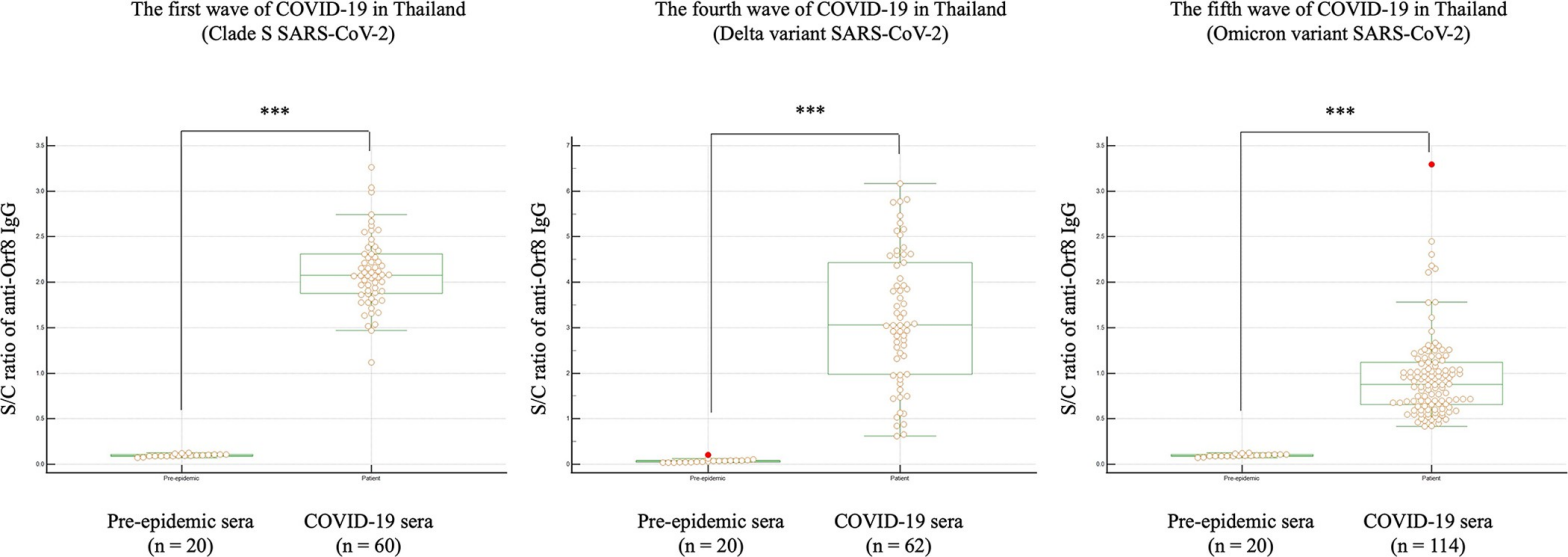
Distribution of anti-Orf8 IgG antibody levels determined in the samples of Thailand’s first, fourth, and fifth waves of the COVID-19 outbreak compared with pre-epidemic sera samples. The graph shows dot plots representing the S/C of the individual samples. The red dot represents the outliner. *** indicates a statistically significant difference at a *p*-value < 0.0001.

**Fig 2:**
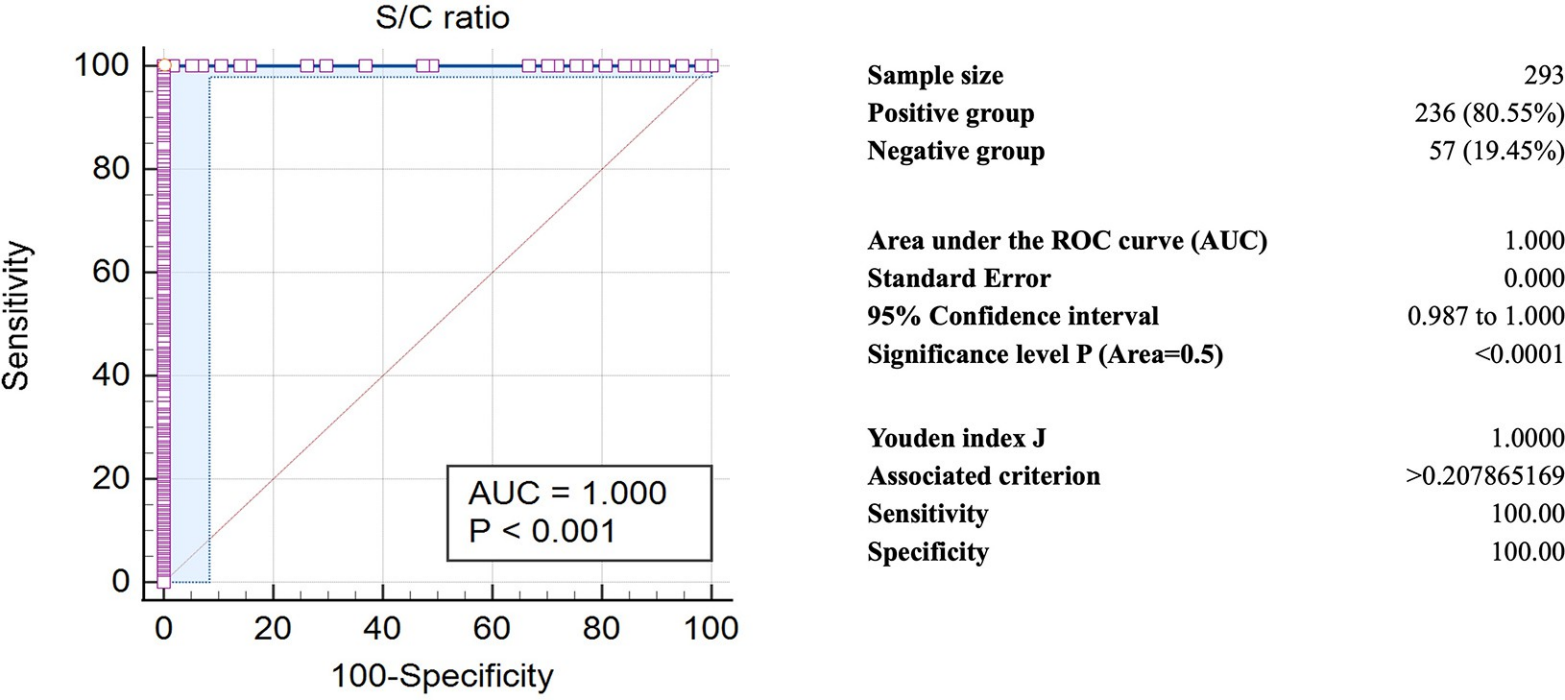
Receiver operating characteristic (ROC) curve of the anti-Orf8 IgG antibody testing of samples collected from three waves of COVID-19 outbreaks.

Antibody response to the Orf8 accessory protein of SARS-CoV-2 in plasma or serum samples collected in Thailand’s first, fourth, and fifth waves of COVID-19 outbreaks was determined using indirect ELISA. The COVID-19-positive samples of the fifth wave were randomly selected from the collection (114 of 327 samples). Pre-epidemic serum samples were negative for neutralizing antibodies against surrogate SARS-CoV-2 viruses (S1 Table). The OD values of all samples were compared with those of the calibrator and expressed as a ratio of S/C signal (S2 Table). IgG antibody response to SARS-CoV-2 Orf8 among patients during the three waves of outbreaks was higher than those of the pre-epidemic sera (Fig 1). The mean ± s.d. of S/C values of sera of the first, fourth, and fifth waves of COVID-19 outbreaks were 2.12 ± 0.15, 3.22 ± 2.12, and 0.95 ± 0.21, respectively. The mean ± s.d. of S/C values of the respective pairwise pre-epidemic sera were 0.09 ± 0.01, 0.08 ± 0.04 and 0.09 ± 0.01, respectively. Using one-way ANOVA and pair-wise analysis, sera collected from the delta-dominant (fourth wave) epidemic exhibited the highest average level of anti-Orf8 IgG, and of those the omicron-dominant (fifth wave) epidemic was the lowest. ROC curve analysis determined the ratios of S/C of all samples for the cut-off S/C value. The optimal S/C cut-off was found to be less than 0.207 (Fig 2). Then the selected S/C cut-off was used to diagnose the COVID-19-positive samples compared with the pre-epidemic sera samples using S/C values (S2 Table). The diagnostic sensitivity and specificity of the anti-Orf8 IgG ELISA for COVID-19 samples from the first, fourth, and fifth waves of outbreaks were found to be 100%.

**Fig 3:**
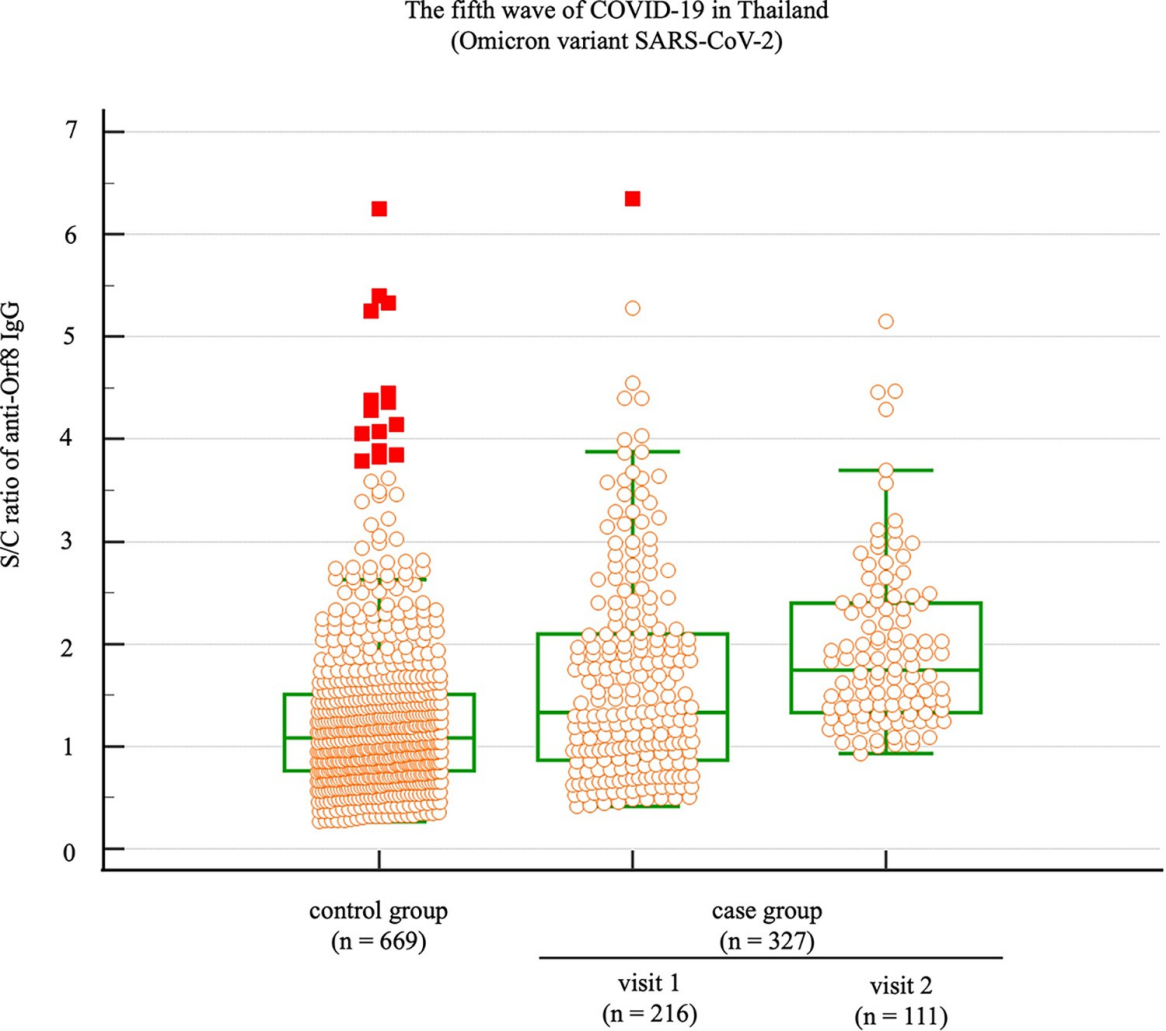
Distribution of anti-Orf8 IgG antibody levels determined in the larger samples collected from patients and the control group during Thailand’s fifth wave of COVID-19. Samples in the case group were divided into two subgroups including samples collected at 7 and 14 days after the RT-PCR positive result called visits 1 and 2, respectively. The graph shows dot plots represent the S/C of the individual samples, while the red squares represent the outliner.

**Fig 4:**
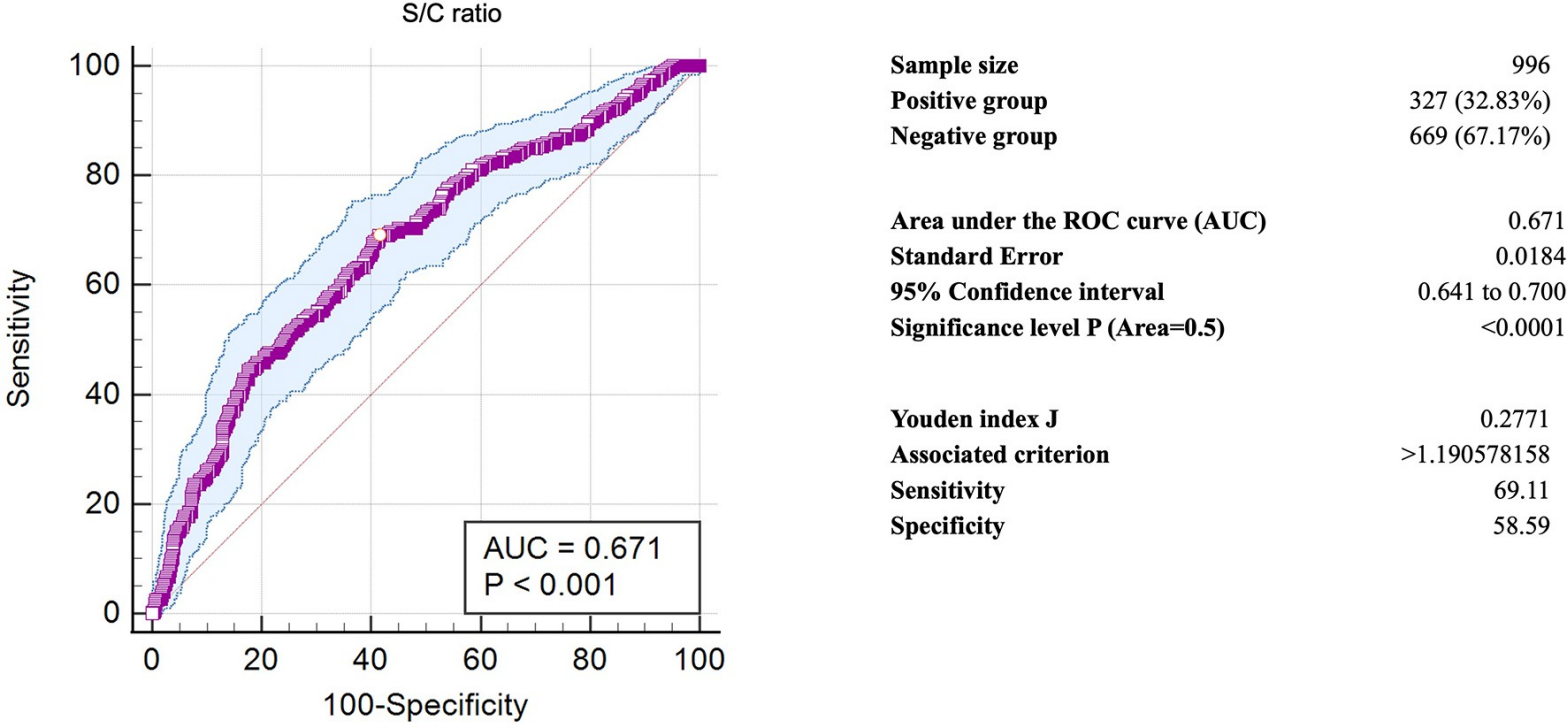
Receiver operating characteristic (ROC) curve of the anti-Orf8 IgG antibody testing of samples collected from the omicron-dominant outbreaks of COVID-19 (the fifth wave).

All COVID-19-positive samples of the fifth wave (327 samples) and control group (669 samples) were tested for anti-Orf8 IgG by the indirect ELISA. Samples of the case group were divided into visit 1 (216 samples) and visit 2 (111 samples) and separately analyzed. The S/C signal ratios were calculated and compared between the case and control groups. The mean S/C between the case and control group did not significantly differ (Fig 3). ROC curve analysis determined the alternative cut-off S/C value from these samples. The optimal cut-off S/C value was higher than 1.19 (Fig 4). Using the alternative cut-off S/C value, the diagnostic sensitivity and specificity of the anti-Orf8 IgG ELISA for COVID-19 samples from the fifth wave of outbreaks were calculated using a 2 × 2 table (Table 1).

**Table 1:** Analysis of diagnostic accuracy of anti-Orf8 IgG ELISA by 2 × 2 table method.

		RT-PCR-positive (Case)		RT-PCR-negative (Control)	
		visit 1	visit 2		
Anti-Orf8 IgG-positive		127 (TP^a^)	99 (TP)	278 (FP^c^)	
Anti-Orf8 IgG-negative		89 (FN^b^)	12 (FN)	391 (TN^d^)	
Visit (V)	Sensitivity (95% CI)	Specificity (95% CI)	PPV^e^ (95% CI)	NPV^f^ (95% CI)	Kappa (95% CI)
V1	58.79%	58.44%	31.36%	81.46%	0.13
	(51.92% to 65.43%)	(54.61% to 62.21%)	(28.36% to 34.52%)	(78.72% to 83.91%)	(0.07 to 0.19)
V2	89.19%	58.44%	26.26%	97.022%	0.24
	(81.88% to 94.29%)	(54.61% to 62.21%)	(24.17% to 28.46%)	(95.01% to 98.24%)	(0.19 to 0.29)

Agreement between real-time RT-PCR and anti-Orf8 IgG ELISA was analyzed by weighted Kappa.

^a^TP = True positive

^b^FN = False negative

^c^FP = False positive

^d^TN = True negative

^e^PPV = Positive predictive value

^f^NPV = Negative predictive value

**Fig 5:**
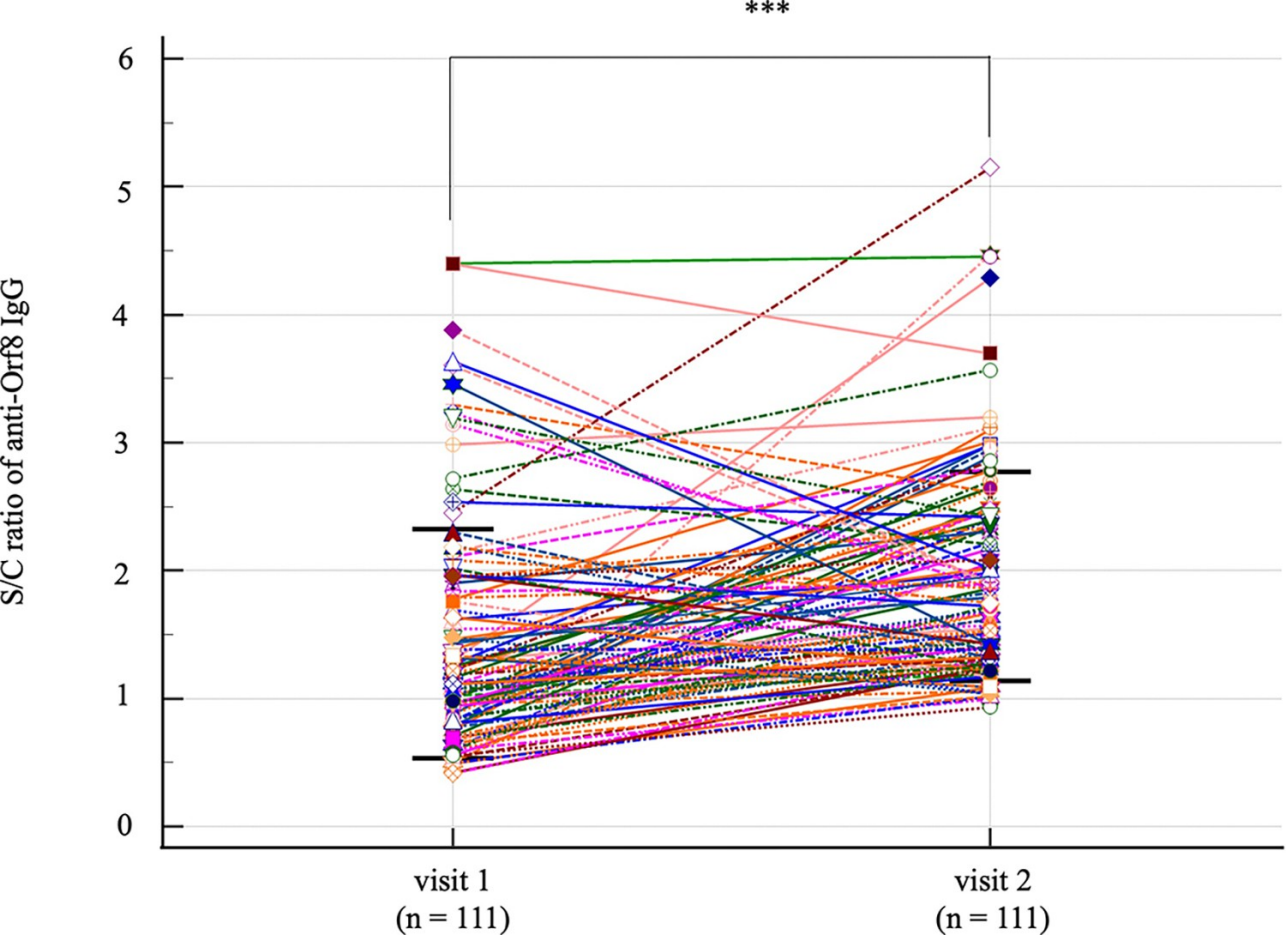
Determination of anti-Orf8 IgG levels in paired samples collected at visit 1 (day 7 after the RT-PCR positive result) and visit 2 (day 14 after the RT-PCR positive result) during the fifth wave of the COVID-19 outbreak. *** indicates a statistically significant difference at a *p*-value < 0.0001.

The diagnostic sensitivity and specificity of anti-Orf8 IgG ELISA for samples of visit 1 were 58.79 and 58.44%, respectively. However, the diagnostic sensitivity and specificity of anti-Orf8 IgG ELISA for samples of visit 2 were improved to 89.91 and 58.44%, respectively. Seven days after the RT-PCR positive result, the anti-Orf8 IgG ELISA showed slight agreement with the RT-PCR as indicated by weighted Kappa at 0.13. The anti-Orf8 IgG ELISA showed a fair agreement with the RT-PCR at 14 days after noting the RT-PCR positive result as indicated by weighted Kappa at 0.24. Paired samples collected from 111 patients with COVID-19 were further analyzed for the levels of anti-Orf8 IgG (Fig 5). The detectable levels of anti-Orf8 IgG on day seven after the RT-PCR positive result were increased from S/C at 1.4287 ± 0.8966 to 1.9555 ± 0.8152 one week later. However, among some patients, anti-Orf8 IgG levels were unchanged or decreased.

### Patient’s background characteristics

Seroprevalence of SARS-CoV-2 among 699 samples from the control group collected during the fifth wave of outbreaks was determined by detecting anti-N IgG. Altogether, 205 samples were collected from the donors who had been fully vaccinated with viral vector or mRNA COVID-19 vaccines which stimulate a serological response toward anti-S antibody production. Among them, 16 samples were positive for anti-N IgG indicating a distinguishable serological response toward vaccination. Nonetheless, 478 samples collected from the donors who had been vaccinated with at least one dose of inactivated whole-virion COVID-19 vaccine tested positive for anti-N IgG. However, in this group, the antibody response is indistinguishable between immunity from natural infection and vaccination. It suggested the background serological response to SARS-CoV-2 among samples in the control group.

## Discussion

Immunological assays to detect SARS-CoV-2 antibodies play an important role in diagnosing recent and past COVID-19 [4]. Data on the seroprevalence of SARS-CoV-2 are essential for estimating disease burden and population immunity. Current serological testing for COVID-19 aims to measure antibodies, either IgM or IgG, specific to viral proteins including S and N. Even though detecting anti-S and anti-N antibodies has shown high diagnostic accuracy for COVID-19, it could not distinguish between antibodies generated in response to vaccine and infection. Orf8 is an immunodominant antigen and an anti-Orf8 antibody has been reported to be an accurate serological marker of SARS-CoV-2 [17]. Orf8 is one of the accessory proteins of SARS-CoV-2 whose amino acid sequence is conserved in SARS-CoV-2 and has low homology to SARS-CoV-1 and common human coronaviruses. Consequently, the Orf8 could be a candidate for use as an antigen of COVID-19 antibody testing [17].

This study demonstrated that all serum samples collected from patients with COVID-19 during Thailand’s first, fourth, and fifth waves of outbreaks revealed anti-Orf8 IgG. It indicated that the SARS-CoV-2 Orf8 protein was highly immunogenic to the sera of patients with COVID-19 conforming to the related results of published papers [16, 17]. Pre-epidemic sera were collected from Songkla Province, Thailand in January 2020, in which local transmission of COVID-19 was limited to Bangkok and most new cases were primarily travel-related. Thus, they were expected for a negligible serological background in ELISA. Compared with pre-epidemic sera, anti-Orf8 IgG ELISA showed 100% diagnostic sensitivity and specificity. Anti-Orf8 IgG could be detected as early as 0 to 14 days and up to 100 days post-symptom onset [17]. In this study, COVID-19 serum samples were collected at 7 to 90 days post-disease onset. Therefore, it could be implied that anti-Orf8 IgG testing could detect SARS-CoV-2 as previously described [17]. Then the anti-Orf8 IgG antibodies were determined in the larger samples collected from patients and the control group during Thailand’s fifth wave of COVID-19. The mean S/C values of anti-Orf8 IgG ELISA signals between cases and controls did not differ. The diagnostic sensitivity and specificity of anti-Orf8 IgG ELISA were poor for diagnosing early SARS-CoV-2 infection at day 7 after an RT-PCR positive result. The sensitivity was significantly improved at day 14 after an RT-PCR positive finding which might have resulted from the rising titer of the anti-Orf8 IgG as demonstrated by analysis of the paired samples. Nevertheless, the specificity did not increase. This might have been due to our inclusion criteria that the control group could not exclude antibody responses to a recent infection. In total, 2.5% of patients in the control group presented anti-N antibodies even though they were diagnosed negative for COVID-19 using real-time RT PCR at the time of sample collection. These patients had been vaccinated with the viral vector and mRNA COVID-19 vaccines. These vaccines were made of nucleotide sequences encoding the S protein of SARS-CoV-2. Hence the detected anti-N antibodies among those samples in the control group might have resulted from natural infection but not from vaccination. However, this speculation is inconclusive without a paired sample analysis. The natural immunity to infection among the remaining samples in the control group could not be verified because the antibody responses could not be distinguished from the immunity of their vaccinations. Moreover, it has been reported that the prevalence of COVID-19 at the Thammasat University Hospital during the fourth wave was 33.3% [21]. Inferentially, the prevalence of COVID-19 in the fifth wave was likely higher than during that period. Therefore, it could be suggested that the seropositivity of anti-Orf8 IgG of some samples of the control group was genuine.

It has been reported that SARS-CoV-2 carrying 382-nucleotide deletion (Δ382) in the genome, causes the removal of the entire *orf8* open-reading frame or transcription-regulatory sequence (TRS) which forbids the *orf8* transcription [22]. Our serological data demonstrated that the Δ382 variant might not have predominated during the COVID-19 outbreaks in Thailand. This speculation was not confirmed by genome sequencing data and bioinformatic analysis. It remains unclear whether such a mutation is still evolving and gaining fitness for human-to-human transmission. Therefore, qRT-PCR and other serological tests are still required for COVID-19 diagnosis.

A reverse transcription quantitative real-time PCR (qRT-PCR) is the confirmatory method and a mainstay for diagnosing COVID-19. Even though it owns inherited sensitivity and specificity, qRT-PCR requires well-trained professionals and biosafety laboratory facilities which dampens large-scale diagnostic screening in a community. Serological testing is rapid, inexpensive, not requiring biosafety laboratory facilities, and can be performed as a mass screening diagnosis or surveillance. It has been reported that the most optimal time for SARS-CoV-2 detection by PCR is between 0 and 4 days post-symptom onset [8, 23]. The diagnostic sensitivity of the qRT-PCR drastically drops after 10 to 14 days post-symptom onset concerning reduced active viral shedding [8]. PCR testing performed 14 or more days from symptom onset usually returns a negative result [23]. This is synchronic with a peak of rising titer of the SARS-CoV-2 antibody [8]. The optimal timing of anti-N and anti-S antibody testing is 14 to 21 days postinfection [24, 25]. However, anti-Orf8 IgG is detectable within 0 to 14 days post-symptom onset and has a notable agreement with qRT-PCR results [17, 24]. The detectable levels of anti-N, anti-S, and anti-Orf8 last two to three months after infection [24, 25]. Nevertheless, the seroconversion rate is influenced by the competence of the host’s immune system, the immunogenicity of viral antigens, and the ability of the virus to subvert the immune responses of the host. Our paired samples analysis suggested the heterogeneity of immune response towards Orf8. Even though most patients developed higher levels of anti-Orf8 IgG as the infection lasted two weeks, some showed unchanged or decreased antibody levels. Moreover, the anti-SARS-CoV-2 antibody level is associated with the severity of symptoms [26]. From weighted Kappa, our results suggested slight to fair agreement between RT-PCR and anti-Orf8 IgG ELISA. Therefore, serological testing has been recommended to complement PCR-based testing but has never intended to supplant it. It would suggest that the utility of anti-Orf8 IgG could be used to confirm SARS-CoV-2 infection, study the epidemiology of COVID-19, and assess vaccine efficacy. Patients with COVID-19 and PCR-negative testing could be confirmed by serological testing using anti-Orf8 ELISA [24]. Regarding the high serological background of the control group, a negative control material or calibrator for comparison is difficult to obtain. Consequently, the diagnosis of SARS-CoV-2 infection should be tested in an appropriate fold increase in the titer of anti-Orf8 IgG from a paired sample. Regarding *orf8* genetic instability, genetic alterations affecting Orf8 expression should be monitored to ensure the utility of the anti-Orf8 IgG ELISA. Our study has highlighted the potential utility of anti-Orf8 IgG antibody testing for seroprevalence surveys but still warrants further investigations. The status of infection among subjects must be defined carefully. The cohort study design seems to be appropriate to determine actual infection status and seropositivity. The data from seroprevalence surveys of anti-Orf8 IgG may provide insights into the disease burden and efficacy of current COVID-19 vaccines as well as the humoral immune response to SARS-CoV-2 Orf8. The latter information is helpful for vaccine development by discovering a new viral antigen candidate. Data on population baseline levels of anti-Orf8 IgG should also be collected and analyzed further to optimize the anti-Orf8 IgG ELISA cut-off value. In this study, the limit of detection could not be determined because anti-Orf8 IgG standard material was unavailable. The limitation this study encountered was the lack of cross-reactivity testing with sera from patients with other respiratory viruses and endemic viral diseases in Thailand such as dengue warranting further validation. However, this concern has proven that a unique sequence of the SARS-CoV-2 Orf8 did not cross-react with the sera from patients with closely related human coronaviruses [16]. The diagnostic accuracy of the Orf8 IgG ELISA, especially specificity, warrants further improvement. Furthermore, large-scale, multi-center investigations are still required to validate the anti-Orf8 IgG ELISA results.

## Supporting information

S1 Table Neutralizing antibodies specific to the S protein of SARS-CoV-2 of the first wave samples using surrogate virus neutralization assays. (XLSX)

S2 Table The OD values and a ratio of S/C signal of all samples. (XLSX)
